# Supplementary figures and images for: The origin of biological homochirality along with the origin of life
Source: PLoS Comput Biol. 2020 Jan 8;16(1):e1007592. doi: 10.1371/journal.pcbi.1007592 (PMC6974302; doi:10.1371/journal.pcbi.1007592)

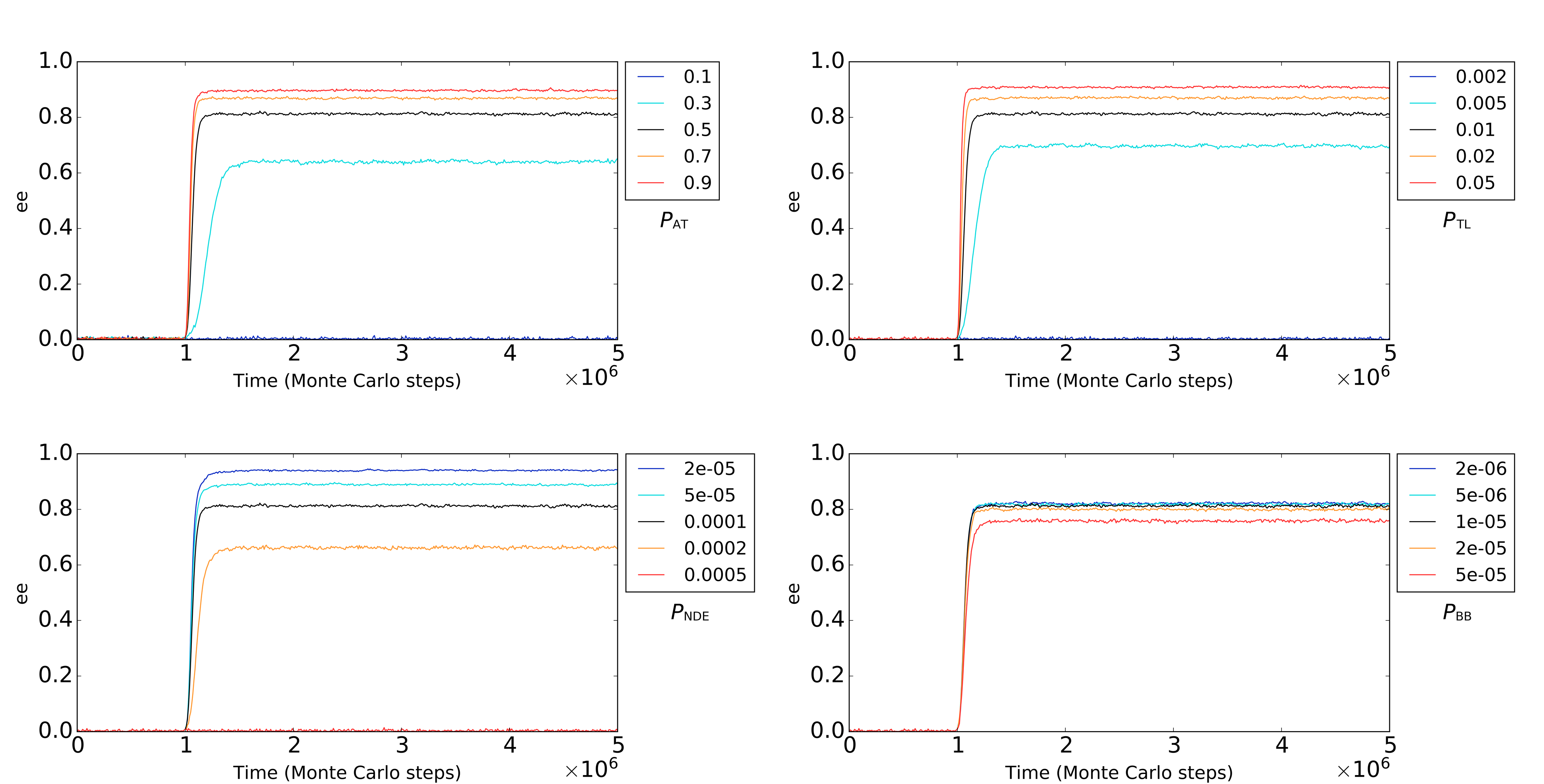

Supplement: S1 Fig — The cases are based upon the solid-line case shown in Fig 2A, i.e., the black-line case in each subfigure is actually identical to the solid-line case in Fig 2A. PAT (probability of an RNA template attracting a substrate) and PTL (probability of the template-directed ligation) are associated with the template-directed synthesis’ efficiency; PNDE (probability of a nucleotide residue decaying at RNA’s chain end) and PBB (probability of a phosphodiester-bond breaking within an RNA chain) are related to RNA’s degradation. (TIF) [file pcbi.1007592.s001.tif]

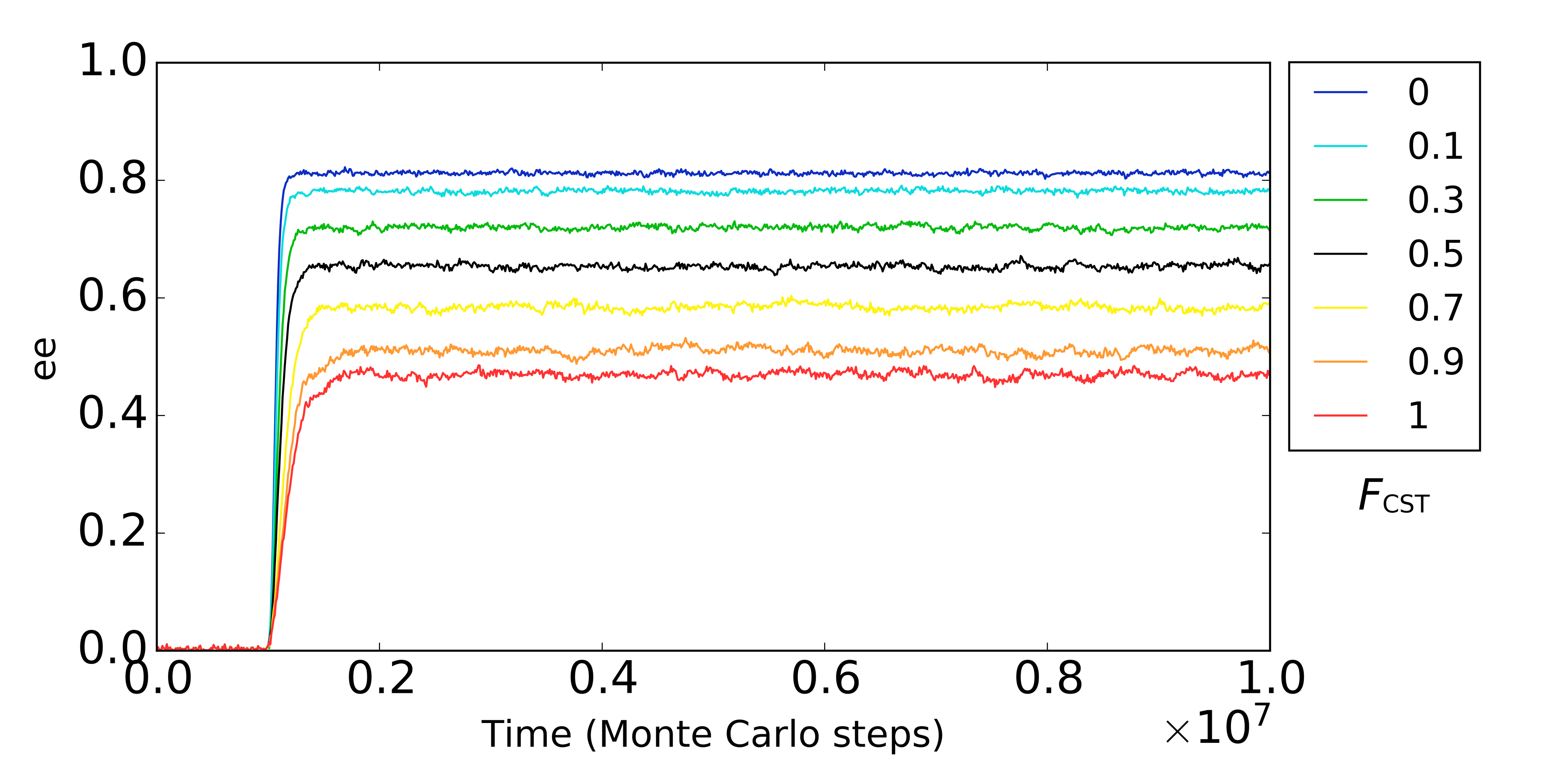

Supplement: S2 Fig — A lower FCST means a higher rate of chiral-selection in the template-directed synthesis. The cases for FCST = 0, 0.5 and 1 have also been shown in Fig 2A, as the solid line, the dashed line and the dash-dotted line there, respectively. (TIF) [file pcbi.1007592.s002.tif]

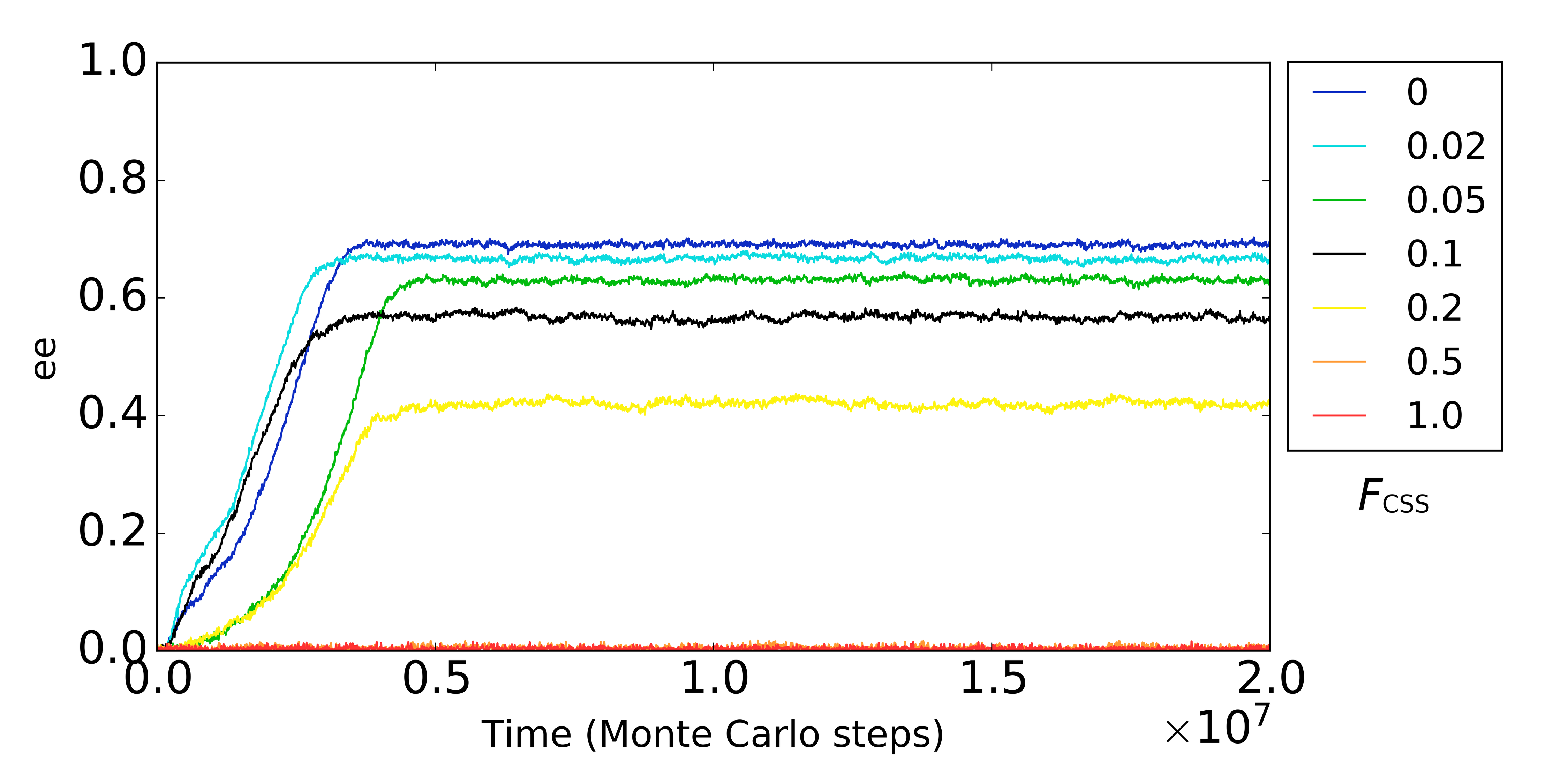

Supplement: S3 Fig — The ee is shown in absolute value, that is, when L-RNA prevails (which type would prevail is actually by chance) and ee is negative, the opposite value is taken–in order to compare all the results in a clear way. A lower FCSS means a higher rate of chiral-selection in the surface-mediated synthesis. The blue-line case in this figure is just the solid-line case in Fig 3. That is, the cases shown here are based on a case in which Rn-mer = R3-mer = 20×R2-mer = 200×R1-mer−note that when considering relevant experiment results, the primer effect could not be so strong [29,30], however, the high level of chirality-deviation in this case provides an ideal base upon which the influence of FCSS can be shown clearly. (TIF) [file pcbi.1007592.s003.tif]
