# Supplementary material for: The origin of biological homochirality along with the origin of life
Source: PLoS Comput Biol. 2020 Jan 8;16(1):e1007592. doi: 10.1371/journal.pcbi.1007592 (PMC6974302; doi:10.1371/journal.pcbi.1007592)
Supplement: Attachment — Submitted filename: response-to-reviewers.docx [file pcbi.1007592.s005.docx]

We thank the reviewers for their comments on our paper. The following is our response to their critiques and suggestions.

**Reviewer #1:**

This paper is an interesting simulation study of the origin of homochirality in the context of the RNA world. I have several comments concerning the reaction scheme in Figure 6.
1. The pink and blue colours of the D and L nucleotides appear as identical shades of grey when the figure is printed in black and white. A dark and light colour would be better. The fact that both D and L monomers are drawn as an L shape is also confusing, particularly when looking at a black and white print.

---Yes, to avoid confusing, we have changed the L shape of monomers to the ‘⊥’ shape (correspondingly, the shape of monomers in Fig. 1 have also been changed), thanks. However, to be consistent with other figures (especially Fig. 5 and Video 1), we tend to keep the colors of D and L nucleotides unchanged. Note that in all these figures, bright colors (which might be observed as deeper colors when printed in black and white) are saved to denote ribozymes with corresponding handedness. In addition, we did not distinguish D and L nucleotides in color-depth is, somehow, to transfer the idea that the two chiral types are actually equivalent in nature.

2. The term surface-mediated synthesis is used to describe random polymerization without a template. There does not seem to be any surface in the model, however. The environment where the random polymerization happens does not have to be a surface, as far as the model is concerned. It could be in solution, or in a lipid membrane system, etc. Therefore it might be clearer to simply call this random polymerization, or non-templated polymerization.

---Yes, here the term surface-mediated synthesis is concerning with random polymerization without a template. In the model, the 2D system can just be thought as a mineral surface, which has been explained in details in our previous work adopting the same kind of modeling system [35,37]. To make it clearer, we have mentioned this point in the revised version (L382-384). In fact, there have long been studies suggesting that the random polymerization of RNA should have occurred on the mineral surfaces (e.g., see [19-22]). Notably, because relevant minerals (particles) could be encapsulated into lipid vesicles, such surface-mediated synthesis of RNA may even have been able to occur in lipid membrane systems [23] – emphasizing the role of surface-mediation in the de novo synthesis of RNA even if the RNA world might have started in a form represented by RNA-based protocells. Corresponding explanations (and two new references [20,23]) has been added into the revised version (L150-156).

3. It takes some time to find the meaning of FCSS and FCST in the text. These are multipliers of the rate of addition of the opposite monomer. This could be shown by writing PRL.FCSS on the lower arrow of each pair, rather than by putting the FCSS in brackets. It is not clear that the two reaction rates are different because the same rate PRL is on both arrows.

---Yes, already modified in the revised version, thanks.

4. It seems natural that chiral selection occurs in the templated case, but I do not understand why the surface mediated synthesis should have chiral selection. Is there any evidence from experiment that there is either slower addition of the wrong monomer or cross inhibition when polymerization is occurring on a surface? There can actually be some chiral crystals of minerals, but I am not sure if this is what is implied in the model. If we simply interpret the 'surface-mediated' reactions as general non-templated reactions, then I don't see a reason for chiral selection.

---Yes, there is evidence from experiments supporting chiral selection and cross inhibition for the surface-mediated synthesis [24-26]. The effect of chiral crystals of minerals on chirality selection of RNA polymerization is not implied in the modelling. In a surface-mediated reaction, it is the residue at the end of a polymer (with the ‘structural’ aid of mineral surface) that favors the incorporation of a monomer with the same handedness as itself – whereas, in the template-directed reaction, both the template and the residue at the end of the polymer may work in the chiral selection. Indeed, if ‘surface-mediated’ reactions are interpreted as general non-templated reactions (e.g., in solution), there seem to be no experimental evidence for the chiral selection – that is, the structural aid of mineral surface is important in the chiral selection of the non-templated reactions. To make it clearer, corresponding explanation and references have been added (L156-159, L172-176).

5. It seems from the diagram that precursors and nucleotides can move across the grid but not polymers. Later in the text it says that polymers can also move according to the Zimm law. It took me a long time to find this. Similarly the diagram appears to say that polymerization is one monomer at a time whereas breakdown can occur in the middle. However, eventually we can find in the text that oligomers of various sizes can also join together. These things are not very clear.

---Yes, we have drawn the movement of polymers in the diagram (see the two stars), and made the corresponding annotation in the legend of the figure (L697-699). Indeed, oligomers of various size can also join into the synthesis, and it is for conciseness that we draw here as if polymerization is one monomer at a time (otherwise the diagram would become very complicated, especially when considering the handedness of residues). We have described the details in the text of Methods, and this annotation have been included in the legend of the figure (L702-704).

The following points are related to my own papers, therefore I will sign my name to the review.
6. The results in Fig 4 show that either the REP or NSR can appear after chirality is established by chiral selection in chemical synthesis. I agree that this makes sense, but I think we should call this chirality arising before life, not chirality arising at the same time as life. I would call the stage involving non-enzymatic templating 'chemical evolution' (as in Tupper et al [18], and Higgs (2017) J Mol Evol 84: 225-235). In the latter paper, I made a point of arguing that chemical evolution is not quite life. Life requires 'biological evolution', which would mean a specific ribozyme sequence like REP or NSR. I would call both the templated and surface mediated reactions chemical not biological. In this case, chirality arises via chemistry, and it is the presence of the chiral bias that allows the specific sequence of the chiral ribozyme to form.

--- According to Wu et al. [9], the arising of homochirality at the same time as the origin of life means that ‘there is no asymmetry in the monomers’ and ‘autocatalytic biopolymers create a chiral bias’. Then, the scenario described here is just such a kind of bio-homochirality origin, considering the autocatalytic feature of template-directed synthesis and surface-mediated synthesis (under the primer effect), as well as the autocatalytic feature of ribozyme-aided reactions. In later papers [18,39], it was elucidated that the polymer synthesis before the emerge of ribozymes should be attributed into the ‘chemical evolution’ and ‘does not quite constitute life’ [18,39]. If so, the present paper is suggesting that the chirality-symmetry breaking occurred at polymer level before the origin of life, and promoted the emergence of initiate functional polymers (with homochirality), which then amplified the chiral bias – during the origin of life. However, if we accept that the origin of life just started with the chemical evolution and then proceeded to biological evolution, and in the sense that the arising and subsequent amplification of the chiral bias as described here just covered such an ‘interface between life and non-life’, it is still quite reasonable to phrase the relevant scenario as ‘the origin of biological homochirality along with the origin of life’. Corresponding explanations have been added into the revised version (L251-266). Thanks. In addition, relevant to this point, we have modified the last sentence of the abstract by adding words ‘at the level of monomers’ (L20-22), in order to emphasize that the significant point here is the origin of homochirality should have occurred along with the advent of biopolymers (from non-functional to functional), rather than somehow at the level of monomers in a chemical background.

Are there any other simulation results in which there is no chiral selection at the chemical stage, but a chiral ribozyme occurs which initiates the chiral bias? This would correspond to chirality arising at the same time as life, in the sense discussed by Wu, Walker and Higgs [9]. I presume that the chiral ribozyme sequence could also form in principle if the polymer synthesis were not chirally biased, but the likelihood of this would be much lower. If there are 4 monomers of one chirality, a ribozyme of length L has a relative frequency of 1 in 4^L, whereas if there are 4 monomers of both kinds, the ribozyme has a relative frequency of 1 in 8^L. The longer the ribozyme, the less likely it is to form before chirality is established. I would suppose that if the ribozyme sequence were much longer than the case of 6 which is shown, then the waiting time between the establishment of the chemical chiral bias and the origination of the ribozyme would be much longer, so it would be clear that life arose after chirality, and it would also be clear that it would be almost impossible for the ribozyme to arise if chirality did not come first.

--- There are not simulation results in which there is no chiral selection at the chemical stage but a chiral ribozyme occurs which initiates the chiral bias (we have not observed such cases in our modeling, and clearly, as the reviewer supposed, if the characteristic sequence were assumed to be longer than 6, the ribozyme would be more difficult to appear). I think that the analysis of the reviewer here about the low possibility of the arising of ribozyme with pure chirality is generally right. However, when the thing is considered in a context of evolution and cross-inhibition is taken into account, things may be somewhat more complicated – therein, because RNA with chimeric handedness residues within the chain is avoided and raw materials are thus ‘saved’, long RNAs with pure handedness may be more likely to accumulate. That is to say, it is likely that the difficulty would not be so great as the reviewer analyzed above. Nevertheless, the reviewer’s anticipation is reasonable – in regard of the modeling here, it is clear that when homochirality is required for a ribozyme, the formation of ribozymes (especially long ones) in chemical evolution would be rather difficult unless a chiral bias could be established by the surface-mediated synthesis and template-directed synthesis as shown in the present study.

7. A final point is that the spatial lattice in this model makes the fluctuations between L and D quite large locally, and this makes it easier to make the instability go one way or the other. This is quite important. It would be worth citing other papers in which chirality arises locally and then spreads across the lattice - e.g. Gleiser & Walker (2009) OLEB 39:479-493; Gleiser & Walker (2008) OLEB 38:293-315.

---Yes, the localizing influence have already been mentioned (L323-332)_and the two references have been cited [47,48]. Thanks.

**Reviewer #2:**

This is a technically sound paper with a limited degree of originality. Consonant with this, the coverage of previous work is rather weak. The whole paper must be reconsidered and rediscussed in the light of missing references.
1. First, I suggest that the Authors put a query to Google Scholar cobnerbing "origin homochirality polymerization model" -- it is easy to find relevant papers, not cited n the present manuscript. I especially advise the authors to look at papers by Brandenburg.

---Yes, we knew these modeling studies (at least some of them) originally (e.g., [9, 41-48]. But these studies only tackled the origin of homochirality at polymer level in a rather abstract way (with a ‘toy model’, as Sandars, the author of the seminal work of this series, called it [41]). For example, they even did not consider template-directed synthesis of the polymers, which played a key role in both chemical evolution (see the comments of reviewer 1) and biological evolution (i.e., Darwinian evolution, based upon molecular replication). In the models, the polymerization runs only in a non-templated way (i.e., the *de novo* synthesis). Another remarkable simplification is that there are not sequence-requirement for the functional polymers, which catalyze the formation of monomers – some of the studies even assumed that all kind of polymers (regardless of lengths) contributed to the overall catalytic efficiency [42-45, 47]. Such assumptions went against general ‘biological senses’ – perhaps because the polymerization concept here is extended from a classic chemical model [49], which address solely a special problem of dimerization (in which the monomers themselves act as auto-catalysts). Therefore, the modellings should be, at least, doubtful in respect of their relevance to reality in the origin of life.

---As a contrast, the present study is based on concrete scenario of the RNA world and ‘formulates’ a detail way towards the establishment of homochirality accompanying the arise of biopolymers – from the roles of surface-mediated synthesis and template-directed synthesis in causing the breaking of the chirality-symmetry to the emergence of homochiral ribozymes (e.g., REP and NSR) and the subsequent chirality-amplification driven by these functional polymers.

---No matter how, with the reviewer’s critique, we see that in the present paper, with a topic concerning ‘origin homochirality polymerization model’, it is unsuitable to neglect these studies (also, Reviewer 1 mentioned the work of Gleiser & Walker [47,48]) In the revised version, we have added a paragraph in Discussion (L303-332) to mention this series of modeling studies and made some explanations about their model (as mentioned above), as well as some comparisons between their results and ours. In this paragraph, the papers by Brandenburg’s group [42-44], as well as others [9,41,45-48], have been cited. Thanks a lot.

2. Even more basic, I suggest taking a close look at this classic:
Pyranosyl-RNA: chiroselective self-assembly of base sequences by ligative oligomerization of tetranucleotide-2’,3’-cyclophosphates (with a commentary concerning the origin of biomolecular homochirality) from the Eschenmoser group.

--- Thanks to the reviewer for bringing this paper to our attention. In this work [50], it was showed that tetramers of pyranosyl-RNA (an RNA-like polymer) containing ‘hemi self-complementary base sequence’ may self-assemble in a chirality-selective way (by ‘cross-templating’) and ultimately give rise to higher oligomers with uniform chirality. The key point is that the original tetramers actually exists as a mixture of all kinds of diastereomers (i.e., most of the tetramers are D-L chimeras). The results led the authors to suggest that biomolecular homochirality may have, in mechanism, originated this way – it was deduced in principle that when the co-oligomerization exceeds a critical level the chirality-symmetry may break. This study is interesting. At least, as also mentioned by the authors [50], it provides a mechanism to the formation of the initiate homochirality template (in respect of the present modeling, as an alternative to the surface-mediated synthesis), which allows the following ‘operation’ of template-directed synthesis in inducing the chirality-deviation. The key doubt is that the substrates are length-fixed (tetramers) and sequence-dependent (hemi self-complementary), which is quite unrealistic. Though the authors have defended for these requirements in some degree, it is not convincing that the scenario in reality may evolve as they anticipated. That is, an important question is that whether such chirality-selective self-assemble of oligomers could still occur when the requirement of their length-fixed and sequence-dependent features is released. It is a typical issue needing corresponding computer modeling studies, which may direct further experimental work. In the revised version, a paragraph is added to discuss this experiment work (all these explanations and comments have been included therein. L333-352).

3. The Authors should carefully reanalyze their results in the light of this paper, and discuss in detail the supposed novelty of their approach.
If it turns out that the message is new only by having an RNA-based model in some detail, but not otherwise, the paper does not reach the publication threshold for Plos Comp Biol.

---Yes, as mentioned above, we have considered carefully the points raised by the reviewer and made comments on the studies mentioned (corresponding citations have been added). We thank the reviewer very much for his critiques and suggestions.

---As explained in our response to point 1, the present study is certainly not one based on the previous series of ‘toy’ modeling studies by Sandars [41], Brandenburg’s group [42-44], and others [9,41,45-48]. Notably, the ‘toy model’ is certainly not a phrase implying contempt (the wording came from Sandars [41], the pioneer of this series of work). In the field of the origin of life, there have been a lot of toy modelling studies, which contributed a lot to our understandings on some important issues. However, in this case, it is also clear that these abstract models are quite insufficient to represent the possible scenario in reality. The abstract models neglected template-directed synthesis completely, and they considered the de novo synthesis but paid no attention to its potential in inducing the chirality-deviation. Perhaps the only content shared between their studies and ours is about the effect of NSR-like functional polymers in promoting chirality-deviation – but they considered such functional polymers in a way going against ‘biological senses’ – for details, see our response to point 1.

--- Notably, beside commenting on the basic message transferred by the experimental work from the Eschenmoser group [50], we have annotated that the authors’ hypothesis based on their results derives a typical issue in which computer modelling may help to solve – this is concerning the role of experimental work and modeling work in the field of the origin of life, which is just the topic of the perspective as summarized in the final paragraph of our paper (in Discussion). A corresponding explanation has been added (L357-360).
